# Supplementary material for: Tightening of tropical ascent and high clouds key to precipitation change in a warmer climate
Source: Nat Commun. 2017 Jun 7;8:15771. doi: 10.1038/ncomms15771 (PMC5467267; doi:10.1038/ncomms15771)
Supplement: Supplementary Information — Supplementary Figures, Supplementary Tables, Supplementary Discussion and Supplementary References [file ncomms15771-s1.pdf]

Supplementary Table 1. **AMIP5/CMIP5 models used in this study.** The corresponding equilibrium climate sensitivity (ECS) and the hydrological sensitivity based on the temperature-mediated precipitation change per unit surface warming ($L_v dP/dT_s$) from the abrupt4×CO_2 simulations are listed. The percentage of precipitation change in parentheses is relative to the multi-model-mean global-mean precipitation of 85.78 W m^{-2}. Missing values are marked '-'.

Model	Modeling Center	ECS in K	Hydrological Sensitivity in W m^{-2} K^{-1} (% K^{-1})
a. BCC_csm1.1*#	Beijing Climate Center, China	2.79	2.14 (2.49)
b. BCC_csm1.1m*#	Beijing Climate Center, China	2.83	2.33 (2.72)
c. CCCMA_canam4/esm2*#	Canadian Centre for Climate Modelling and Analysis, Canada	3.68	2.02 (2.35)
d. CNRM_cm5*#	Centre National de Recherches Météorologiques, France	3.22	2.26 (2.63)
e. CSIRO_access1.0*#	Commonwealth Scientific and Industrial Research Organization - Bureau of Meteorology, Australia	3.78	1.81 (2.11)
f. CSIRO_access1.3*#	Commonwealth Scientific and Industrial Research Organization - Bureau of Meteorology, Australia	3.42	2.17 (2.53)
g. CSIRO_mk3.6*	Commonwealth Scientific and Industrial Research Organization - Queensland Climate Change Centre of Excellence, Australia	4.04	-
h. GFDL_cm3*#	Geophysical Fluid Dynamics Laboratory, USA	3.78	2.21 (2.58)
i. GFDL_esm2g*#	Geophysical Fluid Dynamics Laboratory, USA (no AMIP simulation results)	2.24	1.87 (2.18)
j. GISS_e2r*#	Goddard Institute for Space Studies, USA	2.07	2.67 (3.11)
k. INM_cm4*#	Institute for Numerical Mathematics, Russia	1.97	2.33 (2.72)
l. IPSL_cm5a-lr*#	Institut Pierre Simon Laplace, France	3.94	2.48 (2.89)
m. IPSL_cm5a-mr*#	Institut Pierre Simon Laplace, France	4.02	2.52 (2.94)
n. IPSL_cm5b-lr*#	Institut Pierre Simon Laplace, France	2.56	2.26 (2.63)
o. MIROC_esm*#	Model for Interdisciplinary Research On Climate, Japan	4.60	2.14 (2.49)
p. MIROC_miroc5*#	Model for Interdisciplinary Research On Climate, Japan	2.66	2.33 (2.72)
q. MPI_esm-lr*#	Max Planck Institute, Germany	3.43	2.06 (2.40)
r. MPI_esm-mr*#	Max Planck Institute, Germany	3.29	2.17 (2.53)
s. MRI_cgcm3*#	Meteorological Research Institute, Japan	2.63	2.73 (3.18)
t. NCAR_cam5*	National Center for Atmospheric Research, USA	4.10	-
u. NCAR_ccsm4*#	National Center for Atmospheric Research, USA	2.88	2.34 (2.73)
v. NCC_noresm1-m*#	Norwegian Climate Center (NCC), Norway	2.71	2.26 (2.63)
w. UKMO-hadgem2-a*#	UK Met Office Hadley Climate Center, UK	3.81	1.85 (2.16)

* 23 AMIP5 and coupled historical-RCP4.5 model simulations are analyzed.

\# 21 models are available for the temperature-mediated precipitation response analysis.

Supplementary Table 2. **Observations used in this study and corresponding interannual sensitivities to surface temperature.** All cloud fraction and water vapor sensitivities are for tropical-means and precipitation sensitivity is for global-mean.

Dataset	Analysis Period	Resolution	References	Interannual Sensitivity to T_s
HadCRUT 4.4.0.0 surface temperature	1/1995-12/2005	5°×5°	Morice et al. (2012)Error! Reference source not found.	N/A
CERES EBAF 2.8 fluxes all-sky outgoing longwave radiation	3/2000-10/2015	1°×1°	Loeb et al. (2012)Error! Reference source not found.	3.79 ± 0.41 W m^{-2} K^{-1}
Terra MODIS high cloud fraction Collection 6	7/2002-6/2010	1°×1°	Baum et al. (2012)Error! Reference source not found.	-1.48 ± 0.37 % K^{-1}
Aqua MODIS high cloud fraction Collection 6	7/2002-6/2015	1°×1°	Baum et al. (2012)Error! Reference source not found.	-1.43 ± 0.32 % K^{-1}
Terra-Aqua combined MODIS high cloud fraction, Collection 6	7/2002-6/2010	1°×1°	Baum et al. (2012)Error! Reference source not found.	-1.55 ± 0.36 % K^{-1}
AIRS effective high cloud fraction Version 6	9/2002-12/2013	1°×1°	Kahn et al. (2014)Error! Reference source not found.	-0.68 ± 0.24 % K^{-1}
Joint CloudSat/CALIPSO high cloud fraction	6/2006-6/2015	2.5kmx1.5km	Sassen et al. (2008)Error! Reference source not found. Wang et al. (2012)Error! Reference source not found.	-0.88 ± 2.41 % K^{-1}
ISCCP high cloud fraction	1/1995-12/2005	2.5°×2.5°	Norris and Evan (2015)Error! Reference source not found.	-2.39 ± 0.29 % K^{-1}
Aqua AIRS-Aura MLS upper tropospheric water vapor	8/2004-12/2015	3°×1°	Jiang et al. (2012)Error! Reference source not found.	9.58%$\pm$1.48 % K^{-1}
GPCP Precipitation	1/1995-12/2005	2.5°×2.5°	Huffman et al. (2009)Error! Reference source not found.	2.71 ± 0.90 W m^{-2} K^{-1}
CMAP Precipitation	1/1995-12/2005	2.5°×2.5° source not found.	Xie and Arkin (1997)Error! Re	-0.55 ± 1.04 W m^{-2} K^{-1}

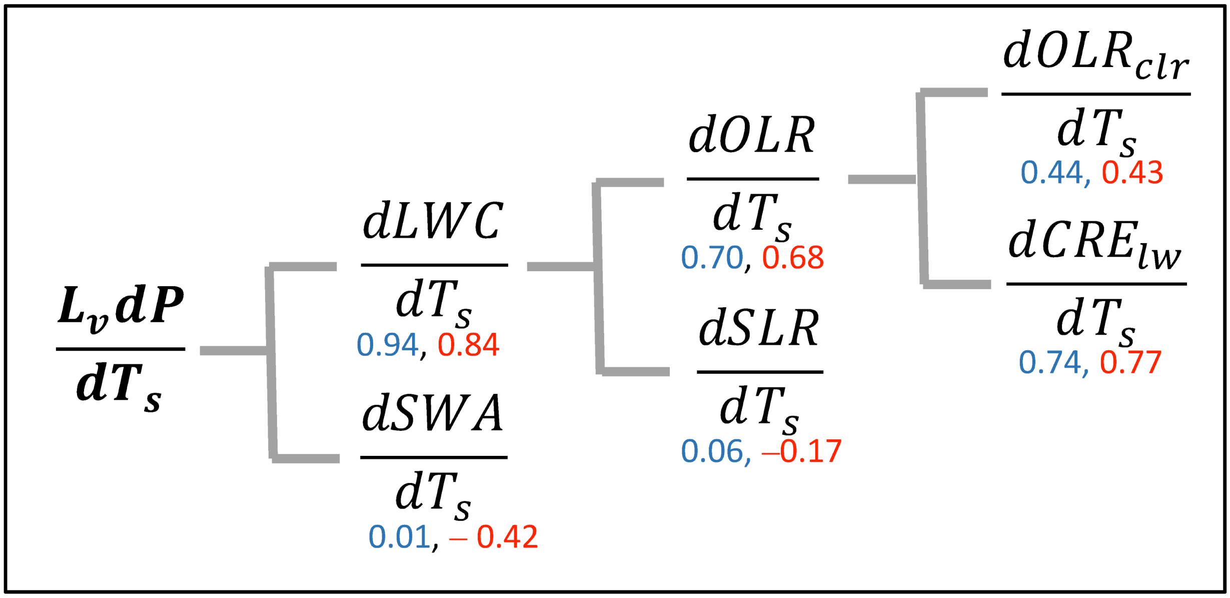

Supplementary Figure 1. Correlations between the inter-model spread in precipitation sensitivity and the inter-model spread in various components of radiative flux sensitivities. The interannual rates are marked in blue and centennial rates are marked in red. All quantities are global-means.

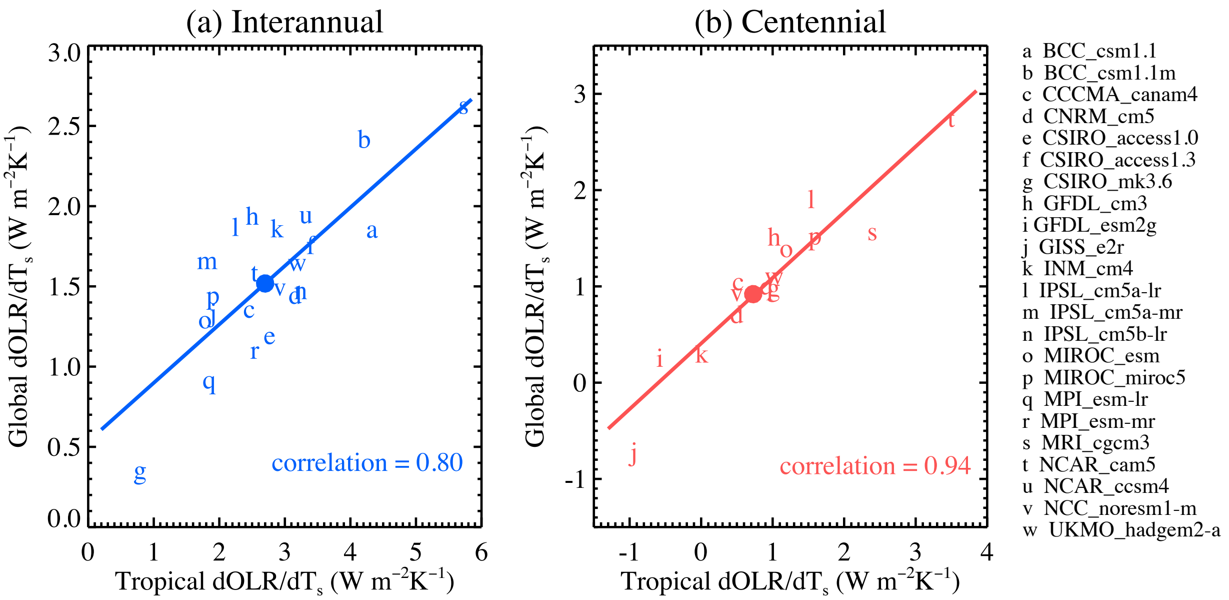

Supplementary Figure 2. Inter-model spread in global-mean OLR sensitivity to surface temperature versus that in tropical-mean OLR sensitivity to surface temperature. (a) interannual (b) centennial. Each model is represented by a lowercase letter. Multi-model-means are marked in solid colored circles. The least-squares linear regression lines and correlation coefficients between the x-axis and y-axis variables are shown.

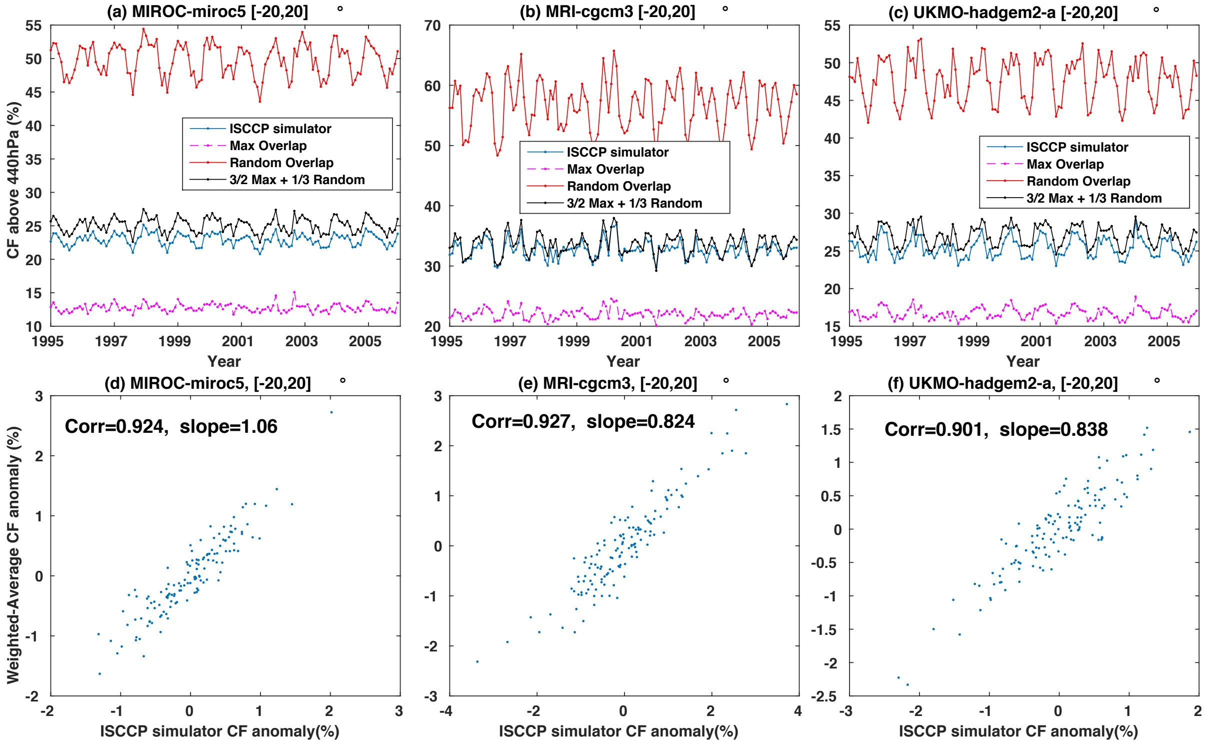

Supplementary Figure 3. Calculating total high cloud fraction using weighted averages under maximum and random overlap assumptions. (a)-(c) The tropical averaged (20°S-20°N) total high cloud fractions based on maximum and random overlap assumptions and the weighted averages of the high cloud fractions under the maximum and random overlap (2/3 maximum and 1/3 random) assumptions, compared to the ISCCP simulator high cloud fractions for three AMIP5 models. (d)-(f) The tropical-averaged de-seasonalized high cloud fraction anomalies based on the weighted averages from the maximum and random overlap assumptions scattered against the ISCCP simulator high cloud anomalies for the period of 1995 to 2005 in the three AMIP5 models.

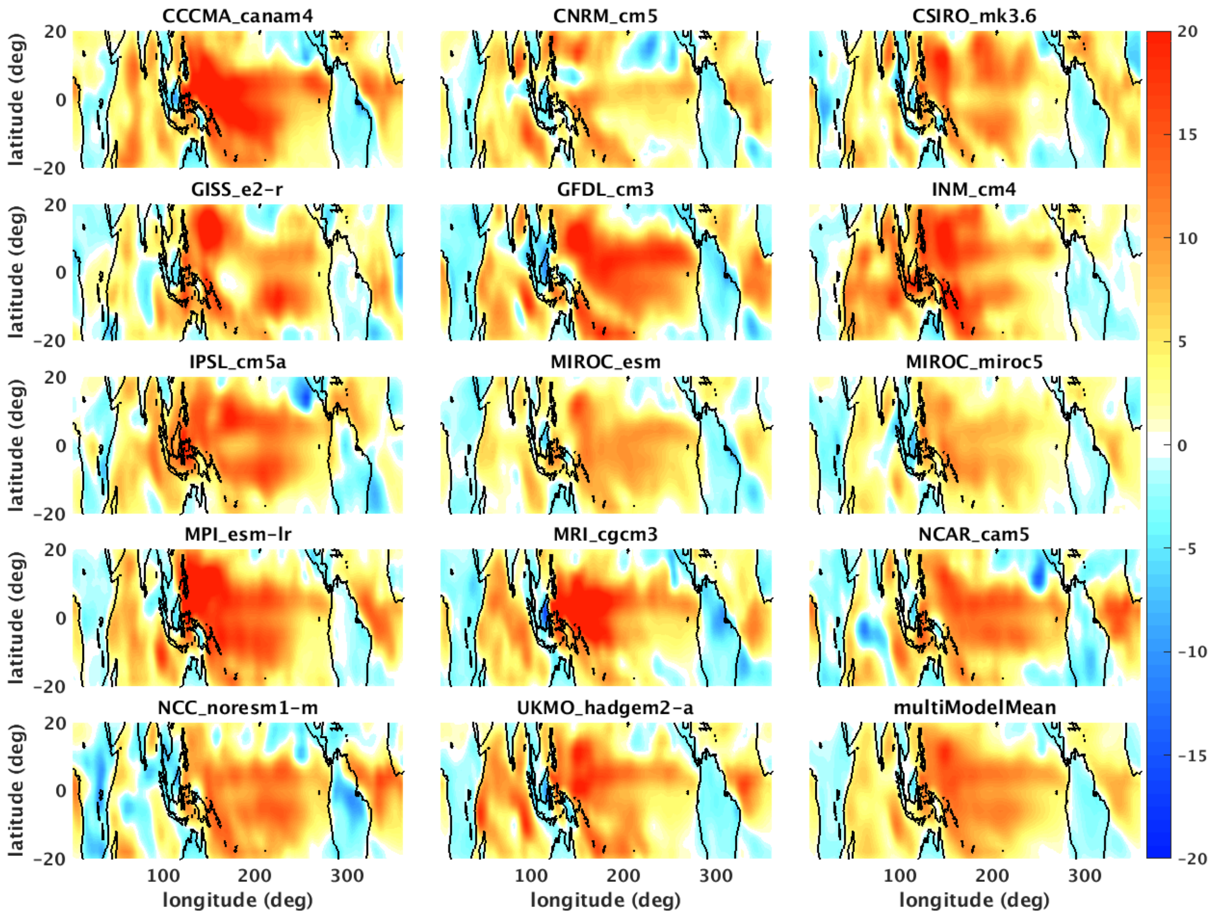

Supplementary Figure 4. Relationship between **interannual high cloud fraction anomalies and local surface temperature anomalies.** The color shadings are the regression coefficients in % K^{-1} for high cloud fraction regressed onto local surface temperature from 1995 to 2005 for 14 AMIP5 model simulations and the multi-model-mean.

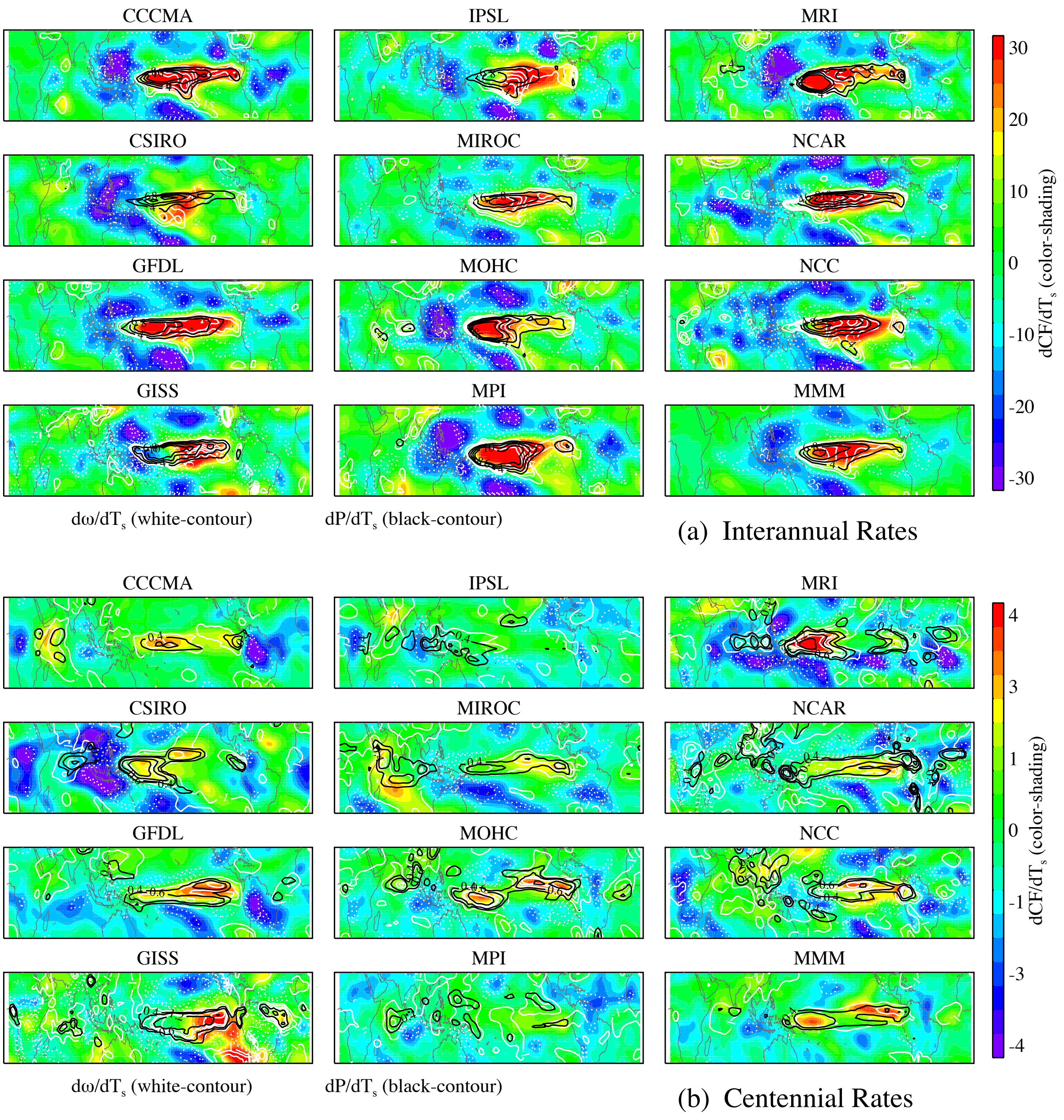

Supplementary Figure 5. Spatial distributions of upper tropospheric vertical velocity at 250 hPa, precipitation and high cloud fraction changes per unit surface warming. (a) interannual rates, represented by the regression slopes of each variable onto tropical-mean (20°S-20°N) surface temperature. (b) centennial rates, represented by the differences between the 21st and 20th centuries for each variable normalized by tropical-mean surface temperature change.

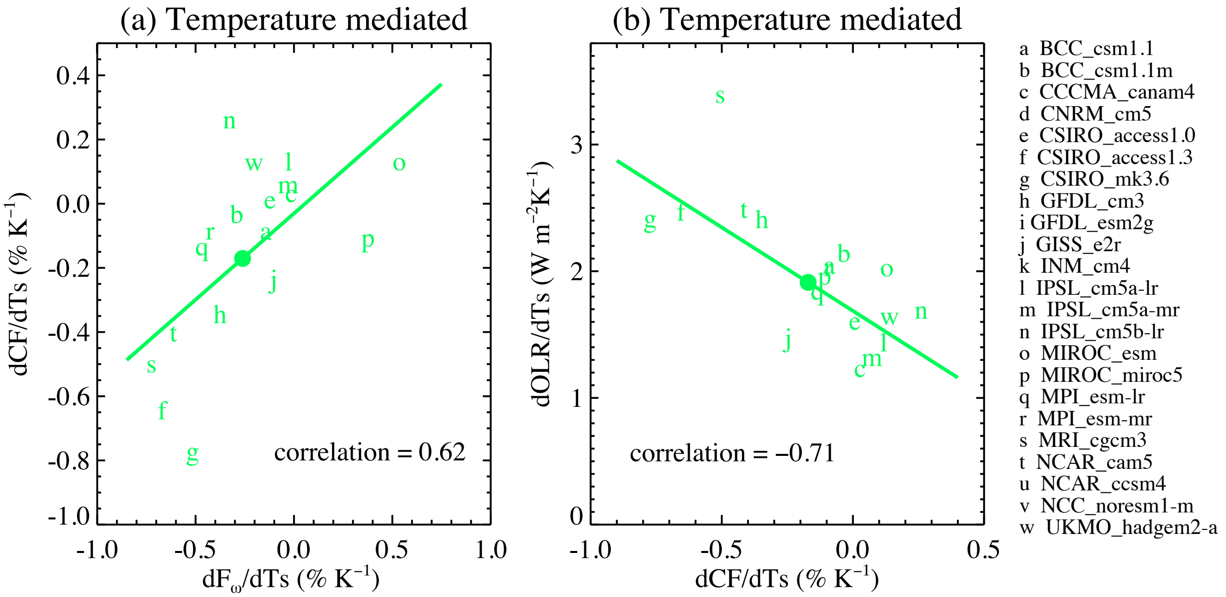

Supplementary Figure 6. Relationships between the inter-model spreads in the tightening of Hadley ascent, the tropical high cloud fraction sensitivity, and the OLR sensitivity for the temperature-mediated rates. (a) Tropical-mean dCF/dT_s scattered against the change of the tropical ascending area fraction per unit surface warming, dF_ω/dT_s. The tropical ascending area is defined by $\omega_{250} < 0$ Pa s^{-1}. (b) The tropical-mean $dOLR/dT_s$ scattered against the tropical-mean dCF/dT_s. The sensitivities are derived from the abrupt4×CO_2 experiments using the linear regression method. Each model is represented by a lowercase letter. Multi-model-means are marked in solid colored circles. The least-squares linear regression lines and correlation coefficients between the x-axis and y-axis variables are shown.

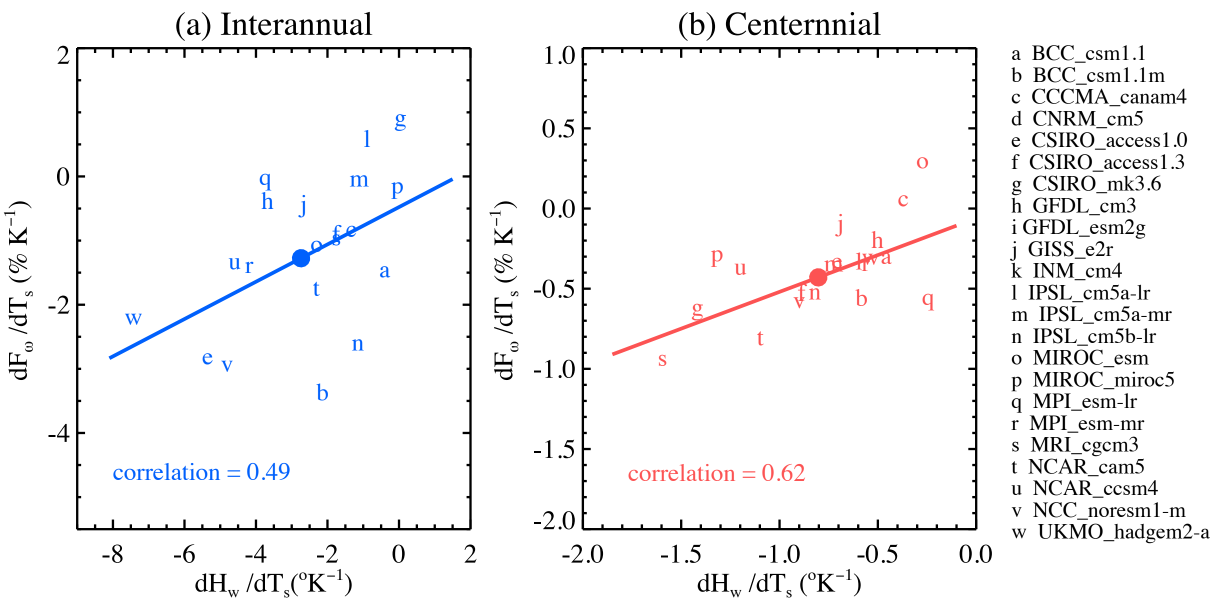

Supplementary Figure 7. Relationship between the change of the width of the ascending branch of the Hadley Circulation and the change of tropical ascending area per unit surface warming. (a) interannual and (b) centennial. The width of the ascending branch of the Hadley Circulation (H_w) is defined by the latitudinal width in degrees of the annual-mean zonal-mean upward vertical velocity at 250 hPa. The change of tropical ascending area per unit surface warming is based on monthly upward vertical velocity at 250 hPa. Each model is represented by a lowercase letter. Multi-model-means are marked in solid colored circles. The least-squares linear regression lines and correlation coefficients between the x-axis and y-axis variables are shown.

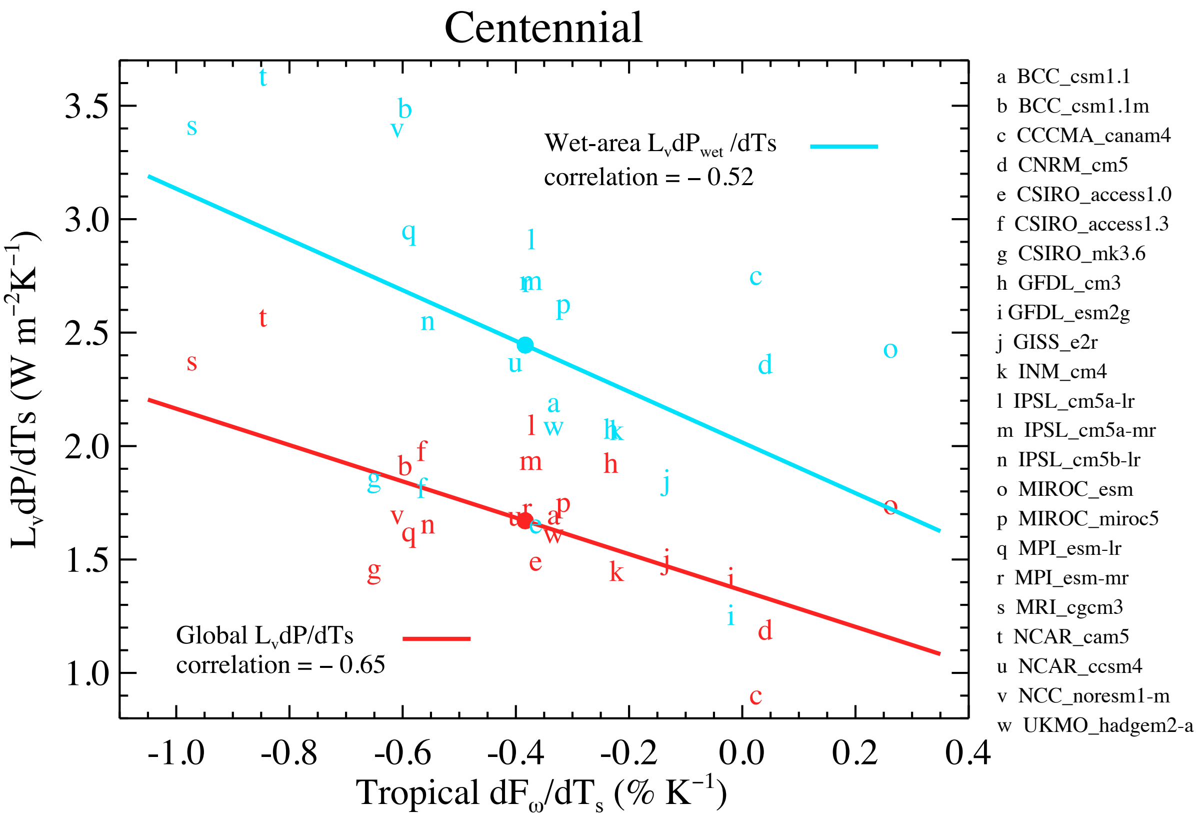

Supplementary Figure 8. Relationships between the tightening of Hadley ascent and precipitation changes. The tightening of the tropical ascending areas based on the upward velocity at 250 hPa (*dF_ω/dT_s*) scattered against the global-mean precipitation change normalized by global-mean surface temperature increase (red) and the tropical wet-area mean precipitation change normalized by tropical-mean surface temperature increase (cyan) for 23 models on the centennial time scale. Each model is represented by a lowercase letter. Multi-model-means are marked in solid colored circles. The least-squares linear regression lines and correlation coefficients between the x-axis and y-axis variables are shown.

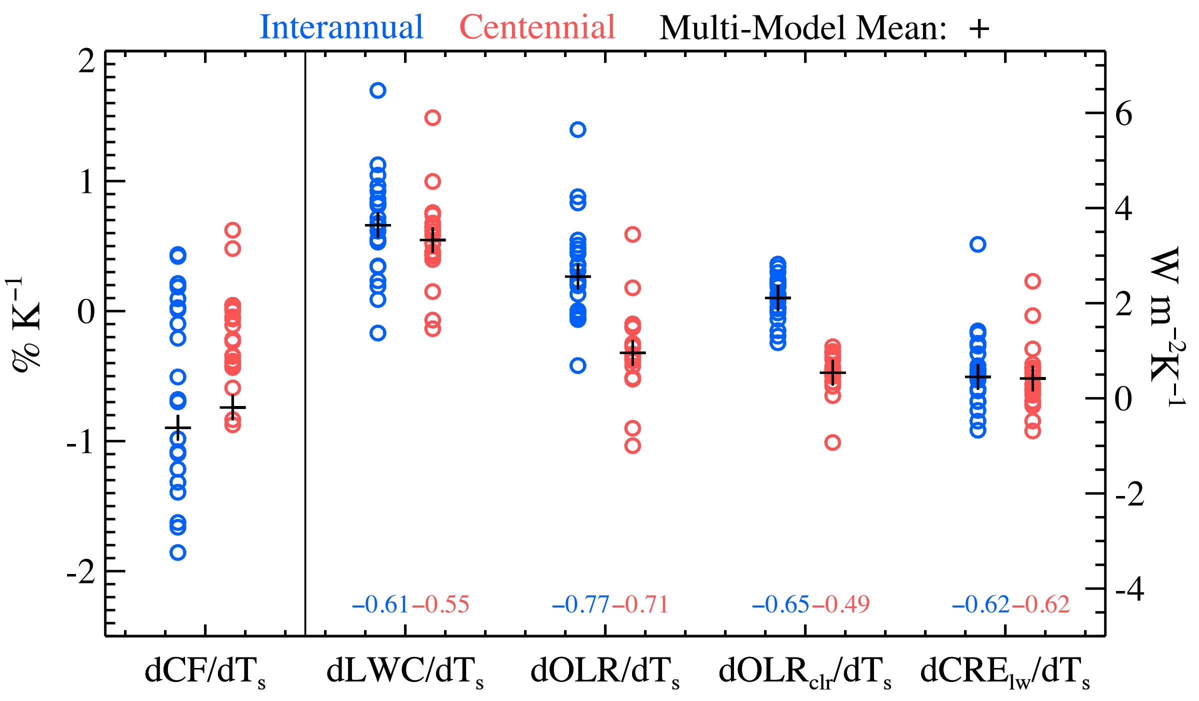

Supplementary Figure 9. Relationships between the inter-model spreads in tropical high cloud fraction sensitivity and longwave radiative sensitivities. Colored circles mark the individual models' interannual (blue) and centennial (red) sensitivities to surface temperature for tropical-mean high cloud fraction (CF), longwave radiative cooling (LWC), all-sky OLR, clear-sky OLR (OLR_{clr}), and longwave cloud radiative effect (CRE_{lw}). Multi-model-means are shown in black "+". The left y-axis is for high cloud fraction sensitivity and the right y-axis is for longwave radiative sensitivities. The across-model correlations between dCF/dT_s and the radiative sensitivities are shown above the x-axis for interannual (blue) and centennial (red) rates.

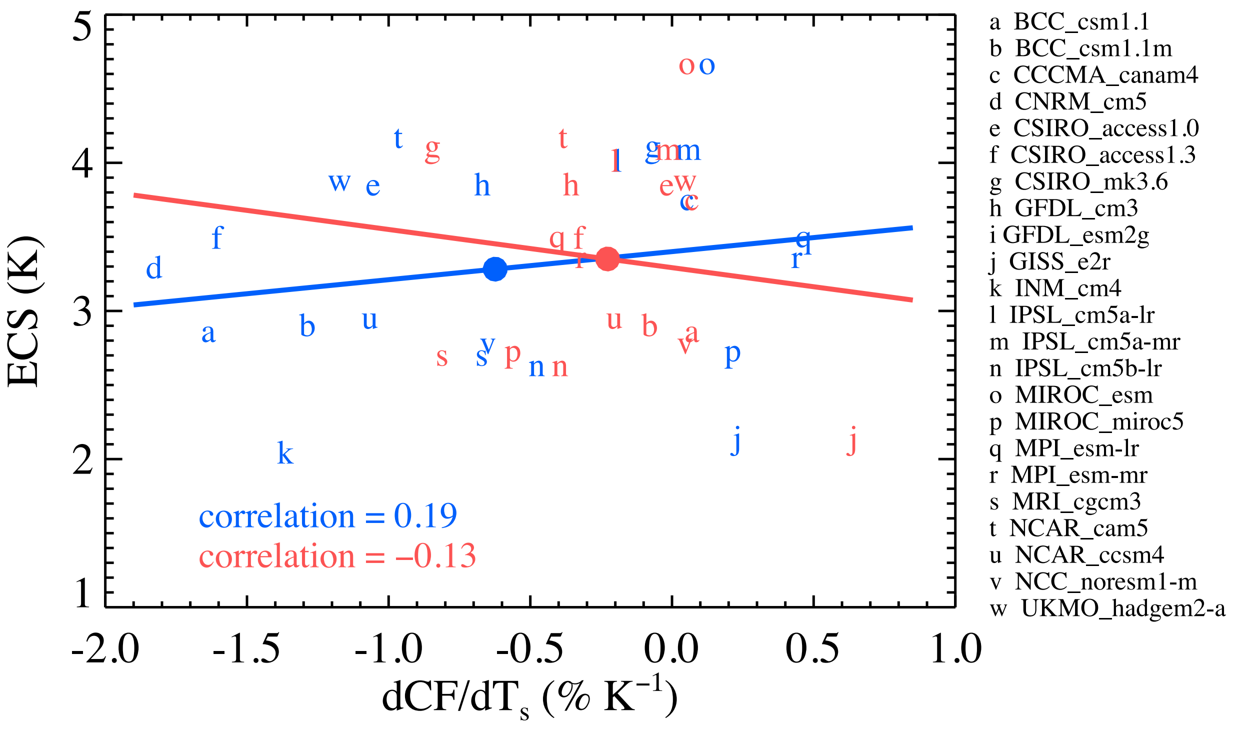

Supplementary Figure 10. Relationship between tropical-mean high cloud fraction sensitivity and equilibrium climate sensitivity. ECS scattered against tropical-mean dCF/dT_s (interannual in blue, centennial in red) across the models. Each model is represented by a lowercase letter. Multi-model-means are marked in solid colored circles. The least-squares linear regression lines and correlation coefficients between the x-axis and y-axis variables are shown.

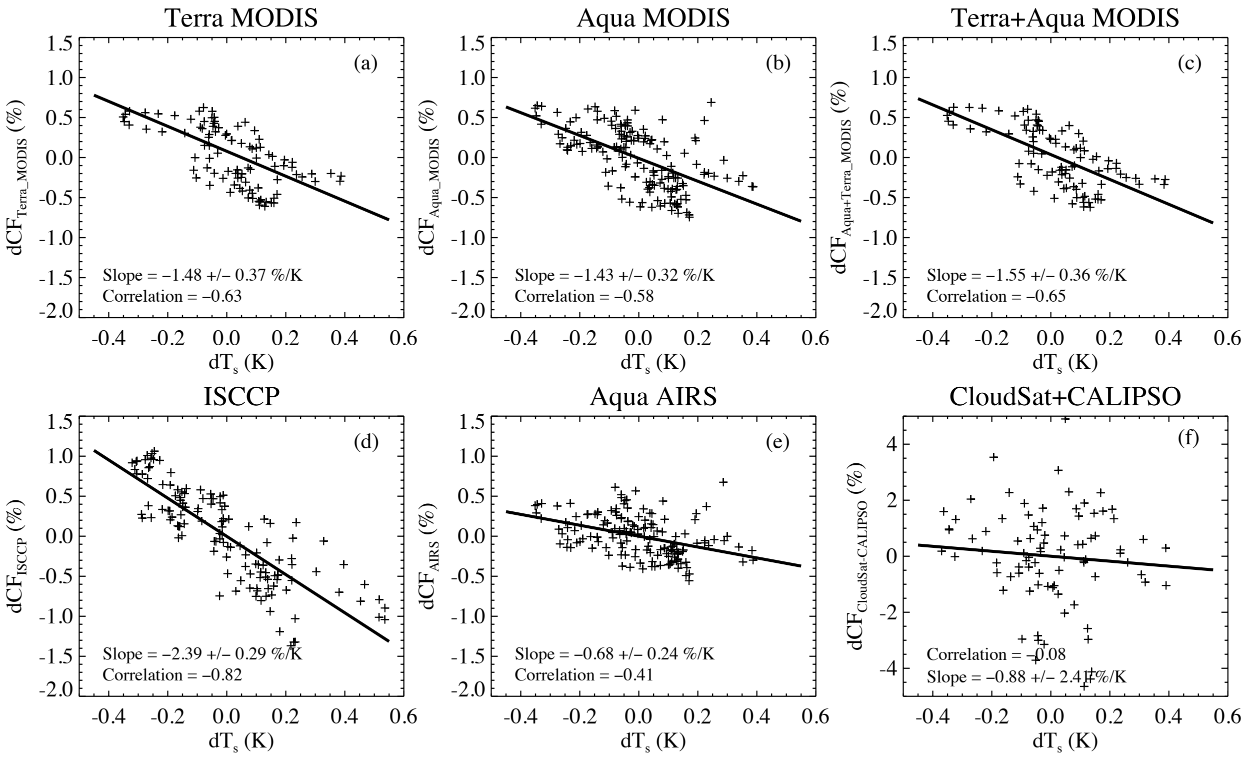

Supplementary Figure 11. **Observed tropical-mean high cloud fraction sensitivity to surface temperature.** (a) Terra MODIS (7/2002-6/2010), (b) Aqua MODIS (7/2002-6/2015), (c) Terra-Aqua MOSIS (7/2002-6/2010), (d) ISCCP (1/1995-12/2005), (e) Aqua AIRS (9/2002-12/2013), and (f) CloudSat/CALIPSO joint retrieval (6/2006-6/2015). The least-squares linear regression lines are drawn, with correlations and regressions slopes marked.

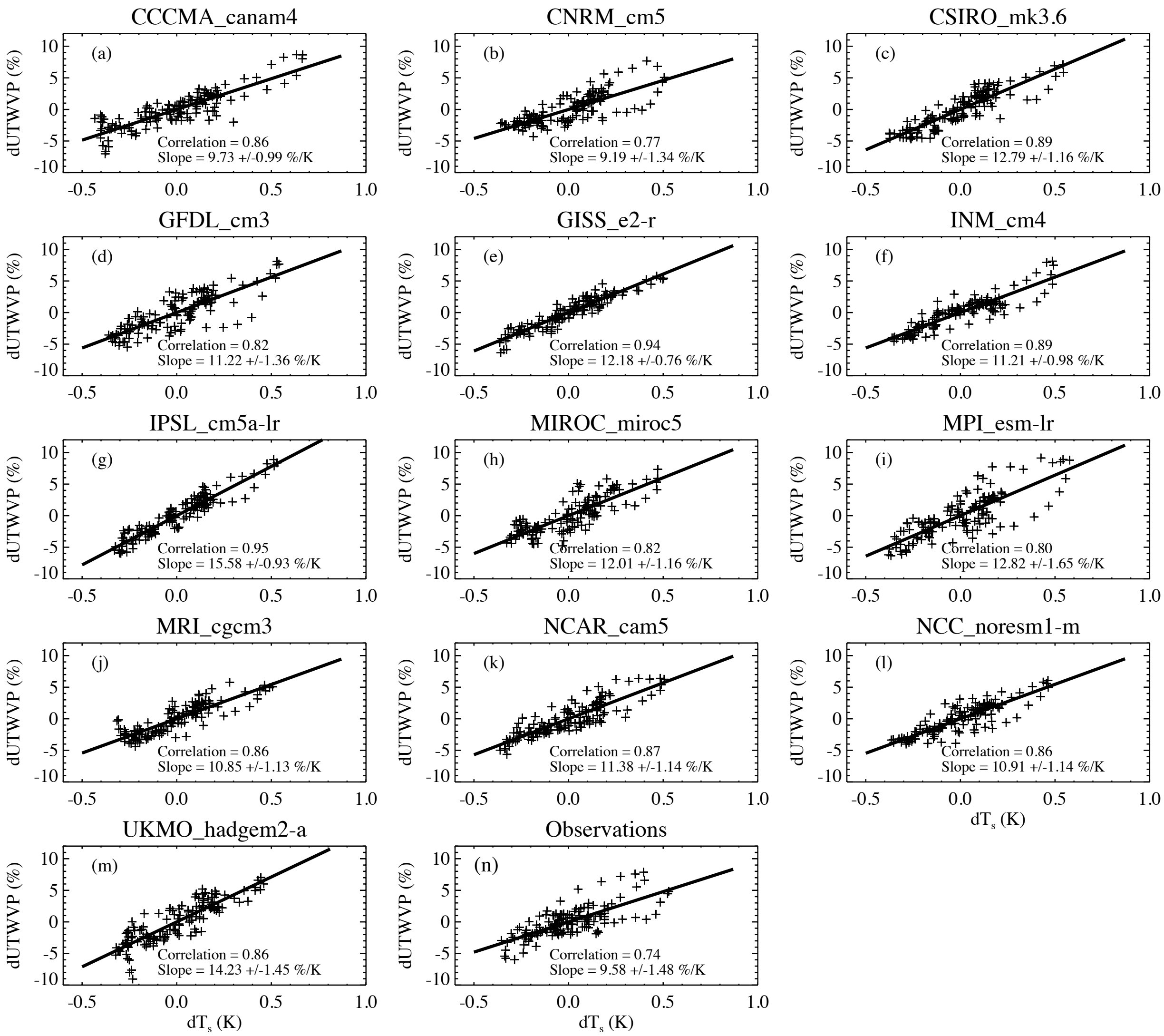

Supplementary Figure 12. **Upper tropospheric (440 hPa to 100 hPa) water vapor path sensitivity to surface temperature in models and observations.** Aqua AIRS and Aura MLS combined water vapor profiles are used for the observed sensitivity. The least-squares linear regression lines are drawn, with correlations and regressions slopes marked.

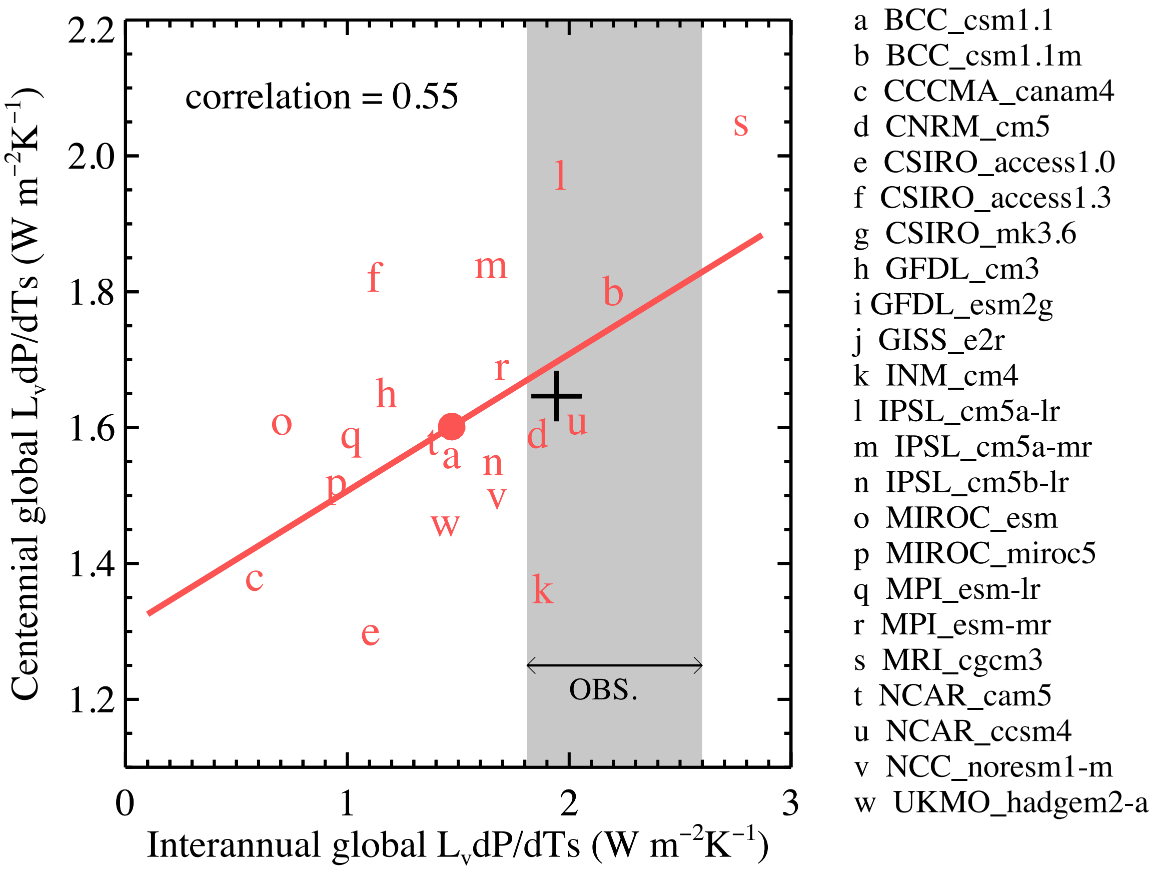

Supplementary Figure 13. Relationship between interannual and centennial global-mean precipitation sensitivity to surface temperature. Centennial $L_v dP/dT_s$ scattered against interannual $L_v dP/dT_s$. Each model is represented by a lowercase letter. The observation-based interannual $L_v dP/dT_s$ with the 95% confidence level is marked in gray shading. The ensemble model means for the 20 models and the five better-performing models are shown in solid circles and black cross, respectively. The least-squares linear regression line and correlation coefficient between the x-axis and y-axis variables are shown.

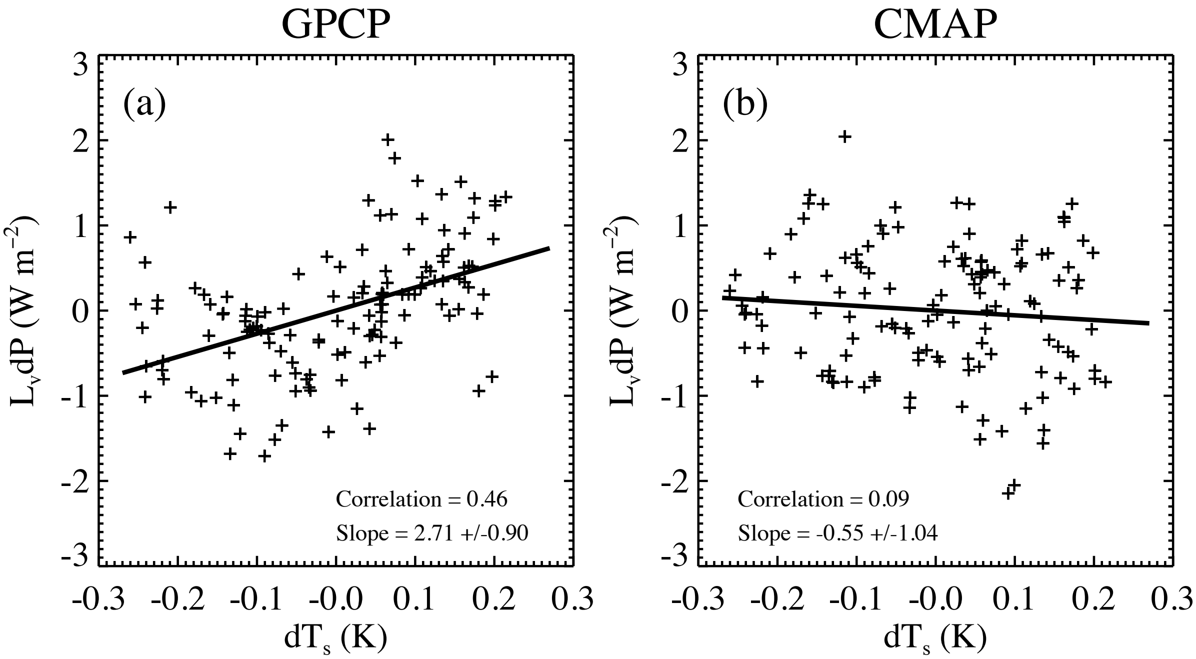

Supplementary Figure 14. Observed global-mean precipitation sensitivity to surface temperature. (a) GPCP and (b) CMAP global-mean precipitation anomalies scattered against surface temperature anomalies for the period of 1995-2005. The least-squares linear regression lines are drawn, with correlations and regressions slopes marked. The CMAP precipitation sensitivity is not used due to large uncertainty.

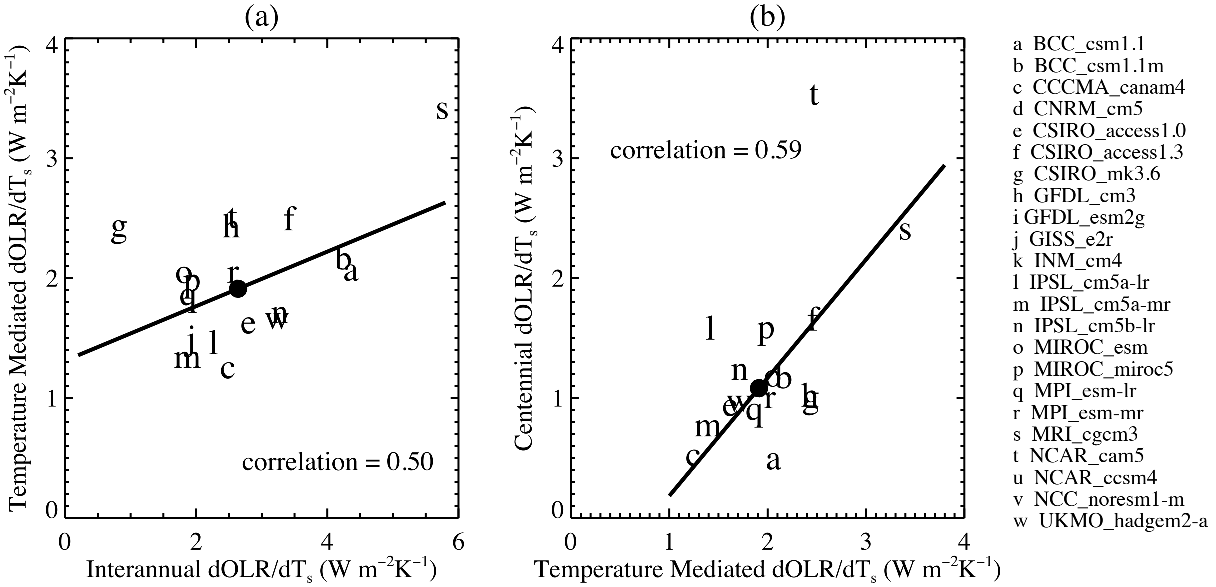

Supplementary Figure 15. Relationships between interannual, temperature-mediated and centennial tropical-mean *dOLR/dT$_s$*. (a) The temperature-mediated *dOLR/dT$_s$* scattered against the interannual *dOLR/dT$_s$*. (b) The centennial *dOLR/dT$_s$* scattered against the temperature-mediated *dOLR/dT$_s$*. Multi-model-means are marked in solid circles. The least-squares linear regression lines and correlation coefficient between the x-axis and y-axis variables are shown.

Supplementary Discussion

The dominance of longwave radiative control on precipitation sensitivity. The dominance of longwave radiation in controlling the global-mean precipitation change is remarkable. We find that the inter-model spread in global-mean dP/dT_s is highly correlated with the model differences in global-mean $dLWC/dT_s$ (R = 0.94 for interannual and 0.84 for centennial rates, Supplementary Figure 1) and the latter is mainly driven by the outgoing longwave radiation (OLR) sensitivity ($dOLR/dT_s$) at the top-of-atmosphere (TOA) (Supplementary Figure 1). Although the atmospheric column LWC is strongly enhanced through increased net downward surface longwave radiation (SLR = surface downward – upward longwave flux) when the surface warms, the model diversity in $dLWC/dT_s$ is not dominated by the differences in $dSLR/dT_s$ (Supplementary Figure 1). Treating OLR as the sum of clear-sky OLR (OLR_{clr}) and longwave cloud radiative effect (CRE_{lw}), we find that the model spreads in $dOLR_{clr}/dT_s$ and $dCRE_{lw}/dT_s$ are both correlated with that in dP/dT_s with larger correlations between $dCRE_{lw}/dT_s$ and dP/dT_s (R=0.74 for interannual and 0.77 for centennial rates, Supplementary Figure 1). Thus, it is reasonable to hypothesize that clouds play an important role in causing the model differences in $dOLR/dT_s$ and therefore $dLWC/dT_s$, which governs the inter-model spread in dP/dT_s.

Model spreads in tropical-mean high cloud fraction sensitivity and longwave radiative sensitivities. Supplementary Figure 9 shows the simulated high cloud fraction sensitivity (dCF/dT_s) in relation to various components of the longwave radiative sensitivities on interannual (blue) and centennial (red) time scales. The rates of dCF/dT_s vary from −1.9% K^{-1} to 0.5% K^{-1} on the interannual time scale for the 22 AMIP models and from −0.87% K^{-1} to 0.62% K^{-1} for the 21 coupled models on the centennial time scale (CNRM_cm5 and INM_cm4 models are missing cloud fraction outputs). The multi-model-means are −0.7% K^{-1} for interannual and

$-0.2\%\ K^{-1}$ for centennial time scales. The inter-model spread in dCF/dT_s is highly correlated with that of $dLWC/dT_s$ on both time scales (correlation $R = -0.61$ and -0.55, respectively), but not with $dSWA/dT_s$ ($R = -0.1$ and 0.05 respectively, figure not shown). The correlations between dCF/dT_s and $dLWC/dT_s$ largely result from the role of high cloud fraction in regulating $dOLR/dT_s$. In comparison, the correlations between dCF/dT_s and $dSLR/dT_s$ are less than 0.3 at both time scales (figure not shown). The inter-model spread in dCF/dT_s is correlated with both model spreads in $dOLR_{clr}/dT_s$ ($R = -0.65$ for interannual and $R = -0.49$ for centennial) and longwave component of cloud radiative effect ($dCRE_{lw}/dT_s$) ($R = -0.62$ for both time scales), suggesting that the amount of high clouds governs not only longwave *CRE*, but also modulates the capacity of clear-sky *LWC* by changing the upper tropospheric dry and clear areas.

High cloud fraction sensitivity in relation to ECS. We find that the correlations between dCF/dT_s and equilibrium climate sensitivity (ECS) are not statistically significant, $R = 0.19$ for the 22 models on the interannual and $R = -0.13$ for the 20 available models on the centennial time scales (Supplementary Figure 10). This indicates that high cloud fraction sensitivity is not a dominant factor in driving the model discrepancy in ECS.

Contribution of upper tropospheric water vapor to the longwave radiative feedback biases. We have analyzed the simulated upper tropospheric water vapor sensitivity to surface warming in the models and find all models capture the increase of upper tropospheric water vapor path (UTWVP) with surface temperature, at the rates between 9.2% K^{-1} and 15.6% K^{-1}, approximately consistent with the Clausius-Clapeyron relation. The multi-model-mean is 11.9% K^{-1}, higher than that derived from the combined AIRS and MLS water vapor observations, 9.6% ± 1.5% K^{-1} (Supplementary Figure 12), although within the AIRS and MLS data uncertainty of ~25%. All models (except CNRM_cm5) produce greater upper tropospheric moistening with

surface warming than the observations. The CNRM_cm5 model has the largest decrease of high cloud fraction with surface warming (Figure 2a) and also the weakest upper tropospheric moistening (Supplementary Figure 12b). The moist biases in the rest of the models, consistent with relatively weak decreases of high cloud fraction (the correlation between the spreads in $dUTWVP/dT_s$ and dCF/dT_s for the 13 models is 0.57), would contribute to the low biases in the magnitudes of $dOLR/dT_s$. However, based on the calculations using the radiative kernels[Error! Reference source not found.,Error! Reference source not found.], we find that the ensemble-mean moist bias of 2% K^{-1} in the upper troposphere would only contribute to a small fraction of the low bias in $dOLR/dT_s$, on the order of 0.05 $W/m^2\ K^{-1}$.

In addition, the inter-model spread in $dUTWVP/dT_s$ has rather weak correlations with the spreads in $dOLR/dT_s$, $dOLR_{clr}/dT_s$ and $dCRE_{lw}/dT_s$ (R = −0.31, −0.23, −0.26, respectively) on the interannual time scale. Therefore, we conclude that the misrepresentation of dCF/dT_s is a dominant source for the model spread in $dOLR/dT_s$ across the CMIP5 models and the moist bias in the upper troposphere associated with the muted high cloud shrinkage contributes only slightly to the low bias in the magnitude of $dOLR/dT_s$.

Determination of the observation-based interannual precipitation sensitivity. Two sets of observations are used to determine the best estimate of the interannual precipitation sensitivity. First, we obtain an OLR-constrained $L_v dP/dT_s$ based on the approximately linear relationship between the model simulated interannual tropical-mean $dOLR/dT_s$ and global-mean $L_v dP/dT_s$ and the CERES observed tropical-mean $dOLR/dT_s$, i.e., the OLR-constrained $L_v dP/dT_s$ = A· $(dOLR/dT_s)_{CERES}$ + B + ε, where A and B are the slope and intercept for the least squares regression across the models, respectively, and ε is the linear fitting residual. The statistical distributions of the slope and intercept for the regression between the modeled $dOLR/dT_s$ and

$L_v dP/dT_s$ are determined by 10000 bootstrap iterations with replacement. The resulting mean slope and intercept are 0.33 and 0.56, respectively. With the CERES $dOLR/dT_s$ at 3.8±0.4 W m^{-2} K^{-1}, the mean value of the OLR-constrained $L_v dP/dT_s$ = 0.33 × 3.8 + 0.56 = 1.8 W m^{-2} K^{-1} with the standard deviation of 0.1 W m^{-2} K^{-1}. Assuming that $L_v dP/dT_s$ contains random variations not captured by the linear relation with $dOLR/dT_s$, the statistics of the fitting residual ε is characterized by all the models' fitting residuals, which yield a standard deviation of 0.39. Thus, the OLR-constrained $L_v dP/dT_s$ has a mean of 1.8 W m^{-2} K^{-1} and a standard deviation of 0.4 = $\sqrt{0.39^2 + 0.1^2}$ W m^{-2} K^{-1}. Hence, the value of the OLR-constrained $L_v dP/dT_s$ at the 95% confidence level (within two times of the standard deviation) is 1.8 ± 0.8 W m^{-2} K^{-1}.

Second, we compute the interannual precipitation sensitivity directly from the least squares regression of the GPCP precipitation onto the HadCRUT4 surface temperature for the period of 1995 to 2005. The 5-month running averaging is applied onto the de-seasonalized anomalies. This gives the GPCP $L_v dP/dT_s$ at 2.7±0.9 W m^{-2} K^{-1}.

Third, we choose the overlapped range of the two observational measures of the interannual $L_v dP/dT_s$, 1.8-2.6 W m^{-2} K^{-1} as the best estimate of the observation-based short-term precipitation sensitivity at the 95% confidence level (Figure 4).

Significance tests for the correlations in the study. For correlation coefficients involving 21 models, the 2-sided student-t test requires $R \geq 0.433$ for the 95% significance level and $R \geq 0.558$ for the 99% significance level. Hence, all the correlations relevant to our conclusions are statistically significant at the 95% level and in many cases at 99% significance level.

Supplementary References

1. Soden, Brian J., Isaac M. Held, Robert Colman, Karen M. Shell, Jeffrey T. Kiehl, Christine A. Shields, Quantifying climate feedbacks using radiative kernels, *J. Clim.*, **21**, 3504-3520 (2008).
2. Shell, Karen M., Jeffrey T. Kiehl, and Christine A. Shields, Using the radiative kernel technique to calculate climate feedbacks in NCAR's Community Atmospheric Model, *J. Clim.*, **21**, 2269-2282 (2008).
3. Morice, C. P., J. J. Kennedy, N. A. Rayner, and P. D. Jones, Quantifying uncertainties in global and regional temperature change using an ensemble of observational estimates: The HadCRUT4 dataset, *J. Geophys. Res.*, 117, D08101, doi:10.1029/2011JD017187 (2012).
4. Loeb, N. G., S. Kato, W. Su, T. Wong, F. G. Rose, D. R. Doelling, J. R. Norris, and X. Huang, Advances in understanding top-of-atmosphere radiation variability from satellite observations. *Surv. Geophys.*, 33, 359- 385. DOI 10.1007/s10712-012-9175-1 (2012).
5. Baum, B. A., W. P. Menzel, R. A. Frey, D. C. Tobin, R. E. Holz, S. A. Ackerman, A. K. Heidinger, and P. Yang, MODIS Cloud-Top Property Refinements for Collection 6. J. *Appl. Meteor. Climatol.*, 51, 1145–1163. doi: http://dx.doi.org/10.1175/JAMC-D-11-0203.1. (2012).
6. Kahn B. H. et al, The atmospheric infrared sounder version 6 cloud products. Atmos. Chem. Phys. 14:399–426. doi:10.5194/acp-14-399-2014 (2014).
7. Sassen, K., Z. Wang, and D. Liu, 2008: The global distribution of cirrus clouds from CloudSat/CALIPSO measurements, *J. Geophys. Res.*, 113, D00A12, doi:10.1029/2008JD009972 (2008).

8. Wang, Z., D. Vane, G. Stephens, and D. Reinke, Level 2 combined radar and lidar cloud scenario classification product process description and interface control document. JPL Rep., 22 pp. (2012).
9. Norris J. R., and A. T. Evan, Empirical removal of artifacts from the ISCCP and PATMOS-x satellite cloud records. *J. Atmos. Ocean. Technol.* 32:691–702. doi:10.1175/JTECH-D-14-00058.1 (2015).
10. Jiang, J. H., et al. (2012), Evaluation of cloud and water vapor simulations in CMIP5 climate models using NASA "A-Train" satellite observations, *J. Geophys. Res.*, 117, D14105, doi:10.1029/2011JD017237 (2012).
11. Huffman, G.J, R.F. Adler, D.T. Bolvin, G. Gu, Improving the Global Precipitation Record: GPCP Version 2.1. *Geophys. Res. Lett.*, 36,L17808, doi:10.1029/2009GL040000 (2009).
12. Xie, P., and P.A. Arkin, Global precipitation: A 17-year monthly analysis based on gauge observations, satellite estimates, and numerical model outputs. *Bull. Amer. Meteor. Soc.*, 78, 2539 – 2558 (1997).
